# Physiological Responses in Relation to Performance during Competition in Elite Synchronized Swimmers

**DOI:** 10.1371/journal.pone.0049098

**Published:** 2012-11-14

**Authors:** Lara Rodríguez-Zamora, Xavier Iglesias, Anna Barrero, Diego Chaverri, Pau Erola, Ferran A. Rodríguez

**Affiliations:** 1 INEFC-Barcelona Sports Sciences Research Group, Institut Nacional d’Educació Física de Catalunya, Universitat de Barcelona, Barcelona, Spain; 2 Departament d'Enginyeria Informàtica i Matemàtiques, Universitat Rovira i Virgili, Tarragona, Spain; University of Bath, United Kingdom

## Abstract

**Purpose:**

We aimed to characterize the cardiovascular, lactate and perceived exertion responses in relation to performance during competition in junior and senior elite synchronized swimmers.

**Methods:**

34 high level senior (21.4±3.6 years) and junior (15.9±1.0) synchronized swimmers were monitored while performing a total of 96 routines during an official national championship in the technical and free solo, duet and team competitive programs. Heart rate was continuously monitored. Peak blood lactate was obtained from serial capillary samples during recovery. Post-exercise rate of perceived exertion was assessed using the Borg CR-10 scale. Total competition scores were obtained from official records.

**Results:**

Data collection was complete in 54 cases**.** Pre-exercise mean heart rate (beats·min^−1^) was 129.1±13.1, and quickly increased during the exercise to attain mean peak values of 191.7±8.7, with interspersed bradycardic events down to 88.8±28.5. Mean peak blood lactate (mmol·L^−1^) was highest in the free solo (8.5±1.8) and free duet (7.6±1.8) and lowest at the free team (6.2±1.9). Mean RPE (0–10+) was higher in juniors (7.8±0.9) than in seniors (7.1±1.4). Multivariate analysis revealed that heart rate before and minimum heart rate during the routine predicted 26% of variability in final total score.

**Conclusions:**

Cardiovascular responses during competition are characterized by intense anticipatory pre-activation and rapidly developing tachycardia up to maximal levels with interspersed periods of marked bradycardia during the exercise bouts performed in apnea. Moderate blood lactate accumulation suggests an adaptive metabolic response as a result of the specific training adaptations attributed to influence of the diving response in synchronized swimmers. Competitive routines are perceived as very to extremely intense, particularly in the free solo and duets. The magnitude of anticipatory heart rate activation and bradycardic response appear to be related to performance variability.

## Introduction

Synchronized swimming (SS) combines swimming, dancing and gymnastics. Swimmers (in solo, duet or team events) perform synchronized routines of elaborate moves in the water accompanied by music. SS became part of the official Olympic program in 1984, initially in the solo and duet modes, was dropped in 1996 in favor of team competition, and was reintroduced in duet competitions at the 2000 Olympic Games. In each program, swimmers competing above junior level must perform both a technical and a free routine. The technical routine is composed of various required elements that are selected every four years. They are performed in a specific order and last 2 min for the technical solo (TS), 2∶20 min:s for the technical duet (TD) and 2∶50 min:s for the technical team (TT). The free routine allows more flexibility to demonstrate grace, artistry and creativity, as there are no figure requirements. Its duration is 3 min for the free solo (FS), 3∶30 min:s for the free duet (FD), and 4 min for the free team (FT) [1].

In modern SS athletes need to combine technically, physically, and esthetically very demanding exercises, lasting about 2 to 4 minutes, performed at increasingly higher levels of intensity both breathing freely and holding breath. Almost 50% of this time is spent in apnea [2]. Consequently, the sport seems to require high levels of aerobic and anaerobic endurance, as well as exceptional breath control when upside down underwater [3]. Most studies on SS have focused on heart rate (HR) and blood lactate measurements after performing single figures [2], [4], [5] or a routine training program [6], [7]. However, barely any of these assessments have been performed during real competition, making it difficult to derive valid information on the physiological demands of the sport and its different events [3].

Information available on the physiological stress during SS is very scarce and several authors have noted the methodological difficulties to assess physiological parameters during SS performance [8]–[11]. Others have suggested that physiological testing in elite SS athletes could help determining the modern demands of this sport [12]–[15]. However, the competitive programs and rules have changed along the years with the addition of acrobatic elements and a greater level of complexity, requiring more speed and power. Today’s competitive demands need to be characterized during real competition, thus requiring a field study design, with some intrinsic limitations imposed by the competition rules on the one side, and by the aquatic environment on the other.

In this context, rates of perceived exertion (RPE) have been shown to be a useful tool for determining exercise intensity, as it is related to physiological indicators of exercise stress, including lactate concentration and HR [16]. The Borg CR-10 category-ratio scale [17] has been recently used to quantify training load in swimming [18] and diving [19]. However, we are not aware of any such research in SS competitions.

Accordingly, the aims of the study were a) to describe the cardiovascular, lactate and perceived exertion responses in junior and senior elite synchronized swimmers during an official competition both in technical and free programs, and b) to relate them with SS performance.

## Materials and Methods

### Study Design

The study was conducted at the 2011 Spanish Absolute Winter Synchronized Swimming Championships. All routines were performed during actual competition with the ad-hoc approval of the Refereeing and Organizing Committees of the RFEN (Royal Spanish Swimming Federation). Most swimmers performed in more than one event, and thus are included in more than one routine group.

### Subjects

Thirty-four female synchronized swimmers, including all swimmers in the Spanish National junior and senior teams–among them Olympic (n = 10), and absolute (n = 4) and junior (n = 7) World Championships medalists–volunteered for the study. They all had competed at national and/or international level at least in the previous two years. Twenty-four were juniors (15–18 years) and ten seniors (>18 years), although they were competing at the absolute National Championships and were not classified according to their age category. The physical characteristics of the subjects are presented in table 1. All subjects voluntarily participated in the study after being informed about the scope and methods of the study, and delivered a written informed consent, with parental permission when needed. The study was approved by the Ethics Committee for Clinical Sport Research of Catalonia.

**Table 1:** Physical characteristics of the subjects.

	All swimmers	Junior	Senior
	(n = 34)	(n = 24)	(n = 10)
Height (cm)	165.2±6.5	163.7±5.1	168.9±8.0*
Body mass (kg)	53. 6±5.6	53.2±5.3	54.6±6.3
Age (years)	17.5±3.3	15.9±1.0	21.4±3.6*
Training (h·week^−1^)	33.1±10.0	29.9±8.2	40.7±10.1*
Years of practice (years)	9.6±2.5	8.7±1.5	11.7±3.3*

Values are mean ± SD.

* Significant differences between senior and junior swimmers (unpaired t-test, P<0.05).

**Figure 1:**
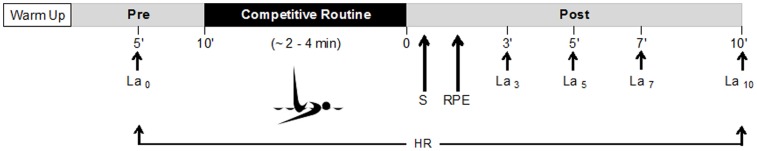
Study protocol. La, blood lactate sample (min); RPE, rating of perceived exertion; S, competition score; HR, heart rate monitoring.

Due to restrictions imposed by the official rules and for ecological validity reasons, we were constrained to monitoring HR, post exercise blood lactate concentration, and RPE during competition. The testing protocol is summarized in figure 1. Routines (n = 96) were performed in a 50-m indoor pool (water temperature: 25–26°C) with 30 m available for use. Prior to each competitive session all swimmers performed 45–60 minutes of general warm-up, including swimming, figures, and monitored routine exercises without music. Additionally, 30 min before their participation, all teams were allotted 20 min of specific rehearsal with music, generally involving the execution of the whole routine and selected parts. HR monitors were placed before the warm up and not removed before 15 min after the routine was executed. Capillary blood samples were taken after warm-up and immediately before the call to perform, and 3, 5, 7, and 10 min after each routine. Every routine was assessed and marked by the official judges of the competition according to FINA rules [1]. The total competition score (TCS) for technical routines is composed of separate scores for execution and overall impression; for free routines the TCS is composed of separate scores for technical merit and artistic impression. In both cases, TCS is up to a maximum of 100 points.

### Heart Rate Monitoring

HR was measured using waterproof HR monitors (CardioSwim, Freelap, Fleurier, Switzerland), which record beat-by-beat HR and lap times using transmitters’ signaling. The belt contains two chest electrodes wired to a monitoring device that can be unloaded on a computer after the recording. Portable beacon transmitters (TX H_2_O, Freelap, Fleurier, Switzerland) were placed by the pool at different locations so that the HR monitors’ microprocessor units could recall specific positions during competition. To minimize potential instrumentation bias, swimmers wore the HR monitor during training sessions within one week before competition. HR was assessed from R–R intervals, 1-s interpolated, and smoothed by computing a running average for 5-s intervals using a 1-s window. HR_pre_ is the average HR for the minute immediately before the start of the routine, after the specific warm-up and following 5-min recovery period; HR_peak_ and HR_min_ are the highest and lowest 1-s value during the exercise, and HR_mean_ is the arithmetic mean for the competitive routine. Post-exercise HR are the average at minutes 1, 3, and 5 (HR_post1_, HR_post3_, HR_post5_).

### Blood Lactate

At every competitive session, following warm-up and a 5-min recovery period and before the call to perform, 10 µL of capillary blood were drawn from the ear lobe. Samples were also taken 3, 5, 7 and 10 min after the routine. Blood samples were immediately analyzed using a lactate photometer (Diaglobal DP100, Berlin, Germany), which had been previously calibrated using lactate standards obtained by dilution (2, 4, 8, 10, 12, 16, and 20 mmol·L^−1^). The highest value was taken as the peak post-exercise lactate concentration (La_peak_).

### Rate of Perceived Exertion

The Borg CR-10 category-ratio scale was selected to rate the perceived intensity of exertion [17]. A graphical, colored, verbal-anchored scale was shown to the swimmers after completing the routine during blood sampling. The week before competition, all participants were given specific instructions on the meaning and use of the RPE scale, and were assessed repeatedly during at least three training sessions so as to disclose learning effects [18] and to improve the consistency of the measurements.

### Video Recording

All routines were continuously recorded using a digital video camera (Panasonic AG-DVX100BE 3-CCD Mini-DV Cinema Camcorder, 50i PAL) at a rate of 50 Hz at a frame rate of 50 fps with an interlaced resolution of 720×576, which allows a time resolution of 0.02 s. For calculations, time values were rounded off to the nearest 0.02 s. The stationary video camera was placed at an elevated site by the pool, just in front of the judges’ podium, and perpendicular to the midpoint of the 30 meters area available for competition. A central computer timer was used for time synchronization of the video and HR and transmitting beacon signals. This was done by filming the timer displayed on the computer screen, and by recording HR monitor activation time on the same computer. Blood sampling was timed using conventional chronographs.

### Statistical Analysis

Descriptive statistics are mean, standard deviation (±SD), minimum value, and range. Differences in HR, La_peak_, RPE, and TCS values were analyzed with a mixed multiple ANOVA for fixed effects and interactions (2 age categories and 6 competitive routines, with Bonferroni correction for multiple pairwise comparisons) and Bonferroni post-hoc tests. Pearson’s correlation coefficients were calculated between all variables for the entire group of swimmers. Stepwise multiple regression analysis was conducted and best predictive models developed (P_in_ = 0.05, P_out_ = 0.10), with TSC (performance) as dependent variable and all physiological parameters as predictor variables. Precise P values are reported and P<0.05 was considered significant.

## Results

Although 96 routines were actually monitored, data collection was complete only in 54 cases, which were those finally included in the statistical analysis; the majority of missing values corresponded to failed or poor quality HR recordings.

### Heart Rate

**Figure 2:**
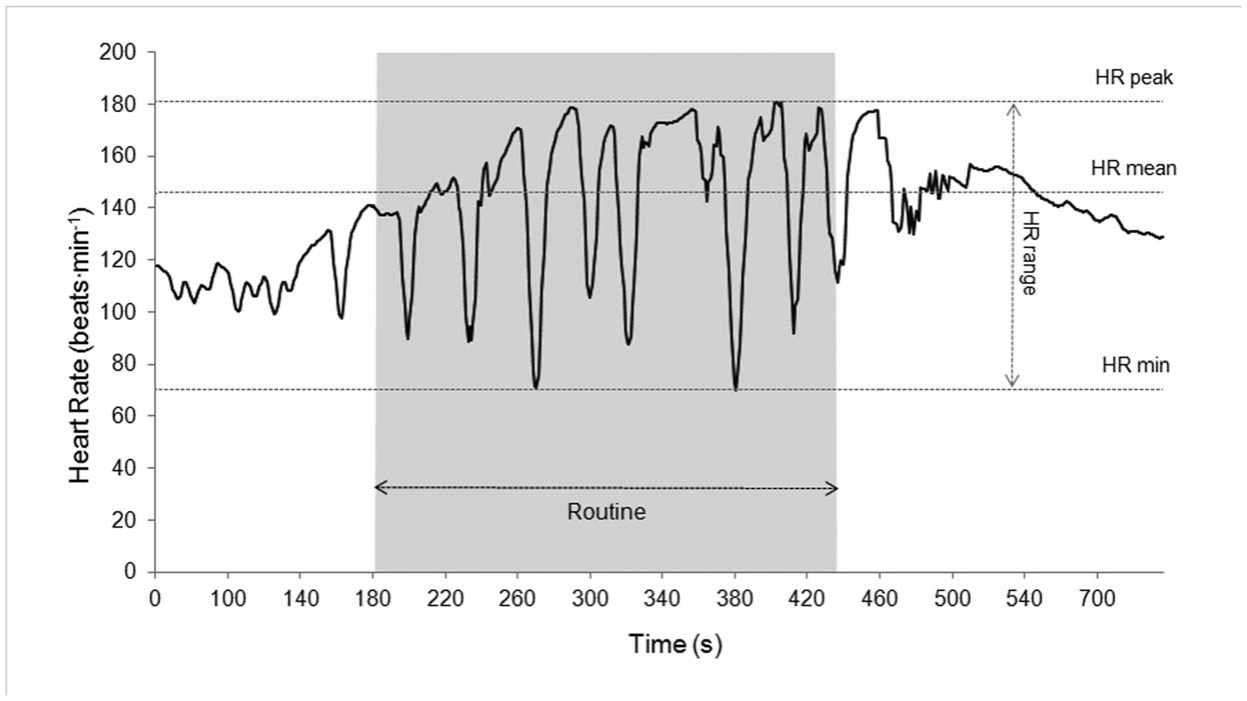
Heart rate profile before, during, and after a competitive free duet routine on an Olympic and World medalist. HR peak, heart rate peak during the routine; HR range, heart rate difference between the minimum heart rate and the maximum value during the routine; HR min, minimum heart rate during the routine; HR mean, the average heart rate during the routine. Line depicts smoothed 5-s averaged values for clarity.

Figure 2 shows an example of a HR response profile before, during, and after a competitive free duet routine on an elite synchronized swimmer (Olympic and World medalist) in which, after a period of anticipatory pre-activation, HR quickly and progressively increases to high levels of tachycardia, interspersed with periods of intense bradycardia during the intense exercise bouts performed while in apnea.

The pattern of HR response during the execution of the six routine programs (table 2) was similar in most of the HR parameters for the entire group of swimmers. However, significant differences were noted in recovery HR (HR_post3_ and HR_post5_) between the TS and the FT routines (P<0.01). Likewise, HR_post3_ was higher in TD as compared to FT routines (P<0.01).

**Table 2:** Heart rate (HR) parameters before (Pre), during (Routine), and after (Post) the competitive routines for the entire group of swimmers.

				TS	FS	TD	FD	TT	FT	All Routines
				(n = 5)	(n = 6)	(n = 10)	(n = 9)	(n = 5)	(n = 24)	(n = 59)
Pre			HR_pre_	122.3±10.8	130.5±15.9	124.6±12.5	130.7±9.6	125.9±10.1	132.0±14.7	129.1±13.1
Routine			HR_peak_	195.5±8.3	189.3±7.6	191.8±10.9	192.5±14.4	192.4±7.3	191.2±5.6	191.7±8.7
			HR_mean_	156.9±9.1	150.1±21.1	161.2±13.1	153.1±20.2	167.2±7.4	162.5±11.6	159.6±14.4
			HR_min_	93.1±21.7	71.4±35.4	94.5±28.1	85.4±27.7	91.2±13.8	89.3±31.3	88.8±28.5
			HR_range_	102.4±17.8	118±34	97.2±25.2	107.1±32.6	101.2±18.8	101.9±31.9	103.5±28.7
Post			HR_post1_	146.6±21.9	146.5±24.1	157.6±12.5	155.3±21.5	155.6±17.0	152.0±35.9	152.7±26.7
			HR_post3_	108.0±12.8	117.8±11.5	113.0±13.7	130.4±7.1	123.1±12.9	128.8±11.0^a,b^	122.9±13.4
			HR_post5_	88.3±18.1	105.1±4.9	103.2±8.3	111.0±15.5	110.5±9.0	113.6±12.2^a^	108.1±13.6

Data are mean ± SD (beats·min^−1^). TS, Technical Solo; FS, Free Solo; TD, Technical Duet; FD, Free Duet; TT, Technical Team; FT, Free Team. HR_pre_, last minute before routine; HR_peak_, HR_mean,_ HR_min_, HR_range_: peak, mean, minimum, and range values during routine; HR_post1_, HR_post3_, HR_post5_: first, third and fifth minutes during recovery. Significant differences (P<0.05) among routine programs were noted only during recovery: ^a^FT vs. TS; ^b^FT vs. TD.

Although HR_pre_ was significantly higher in juniors than in seniors (135.7±10.6 vs. 119.6±10.6 beats·min^−1^, P<0.001), no differences were found within routines for the junior (table 3) or senior (table 4) groups.

**Table 3:** Heart rate (HR) parameters before (Pre), during (Routine), and after (Post) the competitive routines for junior category.

			TS	FS	TD	FD	TT	FT	All Routines
			(n = 2)	(n = 1)	(n = 4)	(n = 8)	(n = 2)	(n = 17)	(n = 34)
Pre		HR_pre_	130. 8±14.2	153.4	135.4±6.1	131.5±9.9	133.8±0.2	137.6±11.5	135.7±10.6
Routine		HR_peak_	202.7±2.7	180.4	189.1±8.0	191.4±14.9	193.3±14.1	190.8±5.0	191.3±9.2
		HR_mean_	158.5±3.0	149.3	164.4±15.4	150.4±19.8	170.2±12.9	166.1±7.4	161.5±13.6
		HR_min_	97.0±12.1	56.5	90.1±25.0	81.9±27.3	84.0±9.8	96.7±26.7	90.5±25.4
		HR_range_	105.8±14.8	123.9	98.9±17.8	109.5±34.0	109.3±23.8	94.1±25.7	100.7±26.1
Post		HR_post1_	135.9±21.0	160.8	167.4±8.2	154.1±22.6	153.1±31.9	158.3±33.8	156.9±27.5
		HR_post3_	98.7±18.5	106.0	118.7±16.7	130.5±7.6	136.7±0.1	129.8±11.7	126.4±14.3
		HR_post5_	74.2±17.4	101.3	108.7±5.2	111.4±16.5	119.2±0.5	113.5±13.8	110.2±15.9

Data are mean ± SD (beats·min^−1^). TS, Technical Solo; FS, Free Solo; TD, Technical Duet; FD, Free Duet; TT, Technical Team; FT, Free Team. HR_pre_, last minute before routine; HR_peak_, HR_mean,_ HR_min_, HR_range_: peak, mean, minimum, and range values during routine; HR_post1_, HR_post3_, HR_post5_: first, third and fifth minutes during recovery.

**Table 4:** Heart rate (HR) parameters before (Pre), during (Routine), and after (Post) the competitive routines for senior category.

			TS	FS	TD	FD	TT	FT	All Routines
			(n = 3)	(n = 5)	(n = 6)	(n = 1)	(n = 3)	(n = 7)	(n = 25)
Pre		HR_pre_	116.7±3.7	125.9±12.5	117.4±10.2	124.4	120.6±9.9	118.4±13.2	119.9±10.6
Routine		HR_peak_	190.6±6.7	191.1±7.0	193.5±12.9	201.5	191.9±2.3	192.1±7.3	192.4±8.0
		HR_mean_	155.8±12.5	150.2±23.6	159.1±12.3	174.7	165.2±3.4	153.7±15.5	156.8±15.3
		HR_min_	90.5±29.0	74.3±38.7	97.4±31.9	113.9	96.1±15.7	71.1±36.1	85.1±32.4
		HR_range_	100.1±22.5	116.7±37.8	96.2±30.8	87.6	95.8±17.7	120.9±39.1	107.3±32.1
Post		HR_post1_	153.7±23.4	143.6±25.8	151.1±10.5	164.8	157.2±7.7	136.5±38.7	147.1±25.0
		HR_post3_	114.2±3.3	121.1±9.2	109.2±11.2	129.5	114.0±4.5	126.0±9.2	118.3±10.7
		HR_post5_	104.9±5.4	99.6±8.2	99.6±8.2	107.3	104.7±5.9	113.9±7.9	105.4±9.4

Data are mean ± SD (beats·min^−1^). TS, Technical Solo; FS, Free Solo; TD, Technical Duet; FD, Free Duet; TT, Technical Team; FT, Free Team. HR_pre_, last minute before routine; HR_peak_, HR_mean,_ HR_min_, HR_range_: peak, mean, minimum, and range values during routine; HR_post1_, HR_post3_, HR_post5_: first, third and fifth minutes during recovery.

### Blood Lactate

For the entire group of swimmers, resting blood lactate was 1.72±0.49 mmol·L^−1^. Maximal values were attained at the 5^th^ or 7^th^ min during the recovery period in all cases. Table 5 summarizes La_peak_ values for each routine program. For the entire group of swimmers La_peak_ was higher in the FS routine (8.5±1.8 mmol·L^−1^) than in the TD (6.8±1.8 mmol·L^−1^, P<0.01) and FT (6.2±1.9 mmol·L^−1^, P<0.001). La_peak_ was also significantly higher in the FD (7.6±1.8 mmol·L^−1^) than in the FT (6.2±1.9 mmol·L^−1^, P<0.01) routines. No significant differences were noted between juniors and seniors (6.7±2.0 and 7.4±2.1 mmol·L^−1^, respectively).

**Table 5:** Peak blood lactate (La_peak_), and rates of perceived exertion (RPE) of the routines.

Category	Variable	TS	FS	TD	FD	TT	FT	All Routines
		(n = 9)	(n = 11)	(n = 16)	(n = 16)	(n = 14)	(n = 30)	(n = 96)
All swimmers	La_peak_ (mmol·L^−1^)	6.9±1.4	8.5±1.8^b^	6.8±1.8	7.6±1.8	7.1±2.4	6.2±1.9^a^	7.3±2.0
	RPE (a.u.)	7.1±1.7	8.0±0.9	7.6±0.9	8.1±0.9	6.6±1.2^d^	7.5±1.1^c,e^	7.0±1.4
Junior	La_peak_ (mmol·L^−1^)	6.1±1.1	8.1±3.3	6.5±1.5	6.9±1.7	7.0±2.7	6.5±1.9	6.7±2.0
	RPE (a.u.)	6.7±1.2	7.4±0.9	8.1±0.6	8.2±0.9	7.4±1.1	7.9±0.8	7.8±0.9*
Senior	La_peak_ (mmol·L^−1^)	7.4±1.5	8.8±1.7	7.0±2.2	8.8±1.4	7.2±2.2	5.3±1.7	7.4±2.1
	RPE (a.u.)	7.3±2.0	8.5±0.5^h^	7.0±0.8	7.8±1.0^i^	5.7±0.5^f^	6.1±1.1^g^	7.1±1.4

Values are mean ± SD. TS, Technical Solo; FS, Free Solo; TD, Technical Duet; FD, Free Duet; TT, Technical Team; FT, Free Team; a.u., arbitrary units (0–10+).

* Significant differences between junior and senior swimmers for all routines. Significant differences among routines in:

La_peak_ (P<0.05) for all swimmers are: ^a^FT vs. FD and FS; ^b^FS vs. TD.

RPE for all swimmers are: ^c^FT vs. FS; ^d^TT vs. FS, TD and FD; ^e^FT vs. FD.

RPE (P<0.05) for the senior group are: ^f^TT vs. TS, FS and FD; ^g^FT vs. FS and FD; ^h^FS vs. TD; ^i^TD vs. TT.

### RPE Score

Mean RPE scores (0–10+) are shown in table 5. For the entire group of swimmers, values for FS (8.0±0.9) and FD (8.1±0.9) exercises were higher than both team routines (FT 7.5±1.1, P<0.05, and TT 6.6±1.2, P<0.01). In both duet routines, FD (8.1±0.9) and TD (7.6±0.9), scores were higher than in TT (6.6±1.2, P<0.01). RPE scores were significantly higher in juniors (7.8±0.9) than in seniors (7.0±1.4, P<0.05). No differences were noted among routines in the junior group. In the senior group, RPE values were higher in the FS routine (8.5±0.5) than in both team routines (FT 6.1±1.1, TT 5.7±0.5, P<0.001). TS (7.3±2.0) and FD (7.8±1.0) elicited higher RPE values than TT (5.7±0.5, P<0.01).

### Total Competition Score

Mean TSC (points) across all routines are presented in table 6. Swimmers attained higher scores in the FT (84.0±4.2) than in the TD (81.0±5.7, P<0.01). Even if this was an absolute competition, seniors (87.0±5 points) were rated higher than juniors (79.1±3.4, P<0.001).

**Table 6:** Total competition score and duration of the competitive routines (time).

Category	Variable	TS	FS	TD	FD	TT	FT	All Routines
		(n = 9)	(n = 11)	(n = 16)	(n = 16)	(n = 14)	(n = 30)	(n = 96)
All swimmers	TCS (points)	81.6±7.2	82.3±7.5	81.0±5.7	82.4±6.6	81.5±5.3	84.0±4.2^a^	82.3±5.7
	Time (min:s)	2∶05±0∶08	2∶58±0∶06	2∶29±0∶06	3∶30±0∶11	3∶00±0∶05	4∶05±0∶06	–
Junior	TCS (points)	75.7±4.5	76.2±3.4	78. 4±3.1	78.0±2.6	78.5±3.7	81.1±2.5^b^	79.1±3.4*
	Time (min:s)	1∶56±0∶10	2∶58±0∶04	2∶27±0∶07	3∶28±0∶09	2∶57±0∶03	4∶04±0∶05	–
Senior	TCS (points)	87.5±4.5	88.3±4.8^c^	85.3±5.7	87.8±6.6	85.8±4.2	88.1±4.5	87.0±5
	Time (min:s)	2∶09±0∶04	2∶58±0∶08	2∶30±0∶06	3∶34±0∶14	3∶03±0∶04	4∶11±0∶06	–

Data are mean ± SD. TS, Technical Solo; FS, Free Solo; TD, Technical Duet; FD, Free Duet; TT, Technical Team; FT, Free Team; TCS, total competition score.*Significant differences between junior and senior swimmers for all routines.

Significant differences (P<0.05) are:

For all swimmers: ^a^FT vs. TD.

Among juniors: ^b^FT vs. TS, FS, TD and FD.

Among seniors:^ c^FS vs. TT.

### Physiological Correlates of Performance

TCS performance scores negatively correlated with HR_pre_ (R = −0.41; P<0.001) and HR_min_ (R = −0.24; P<0.05), and positively correlated with HR_range_ (R = 0.22; P<0.05). In the stepwise multiple regression analysis the best model included only HR_pre_ and HR_min_ (R_m_^2^ = 0.26; P<0.0001; SEE = 4.86). No other significant bivariate or multivariate correlations were found between TCS and the rest of HR, La_peak_, and RPE variables.

## Discussion

To our knowledge, this is the first study in which the physiological responses to SS routines during an official competition in high-level swimmers are characterized. We found a very intense anticipatory HR pre-activation in all swimmers, even more pronounced in juniors. During the execution of all routines in both age-category groups, cardiovascular demands were equally high, with HR quickly approaching maximal levels, and interspersed periods of marked bradycardia during the intense exercise bouts performed in apnea. In contrast, differences were noted among routines in blood lactate levels, with highest values in free solo, followed by free duets and technical and team routines. Both HR pre-activation and bradycardia were moderately related to performance.

### Heart Rate Response

A remarkably high HR pre-activation was observed in all subjects and routines (table 2). This conspicuous HR dynamics alteration before the actual start of the exercise is likely due to: 1) the effect of the previous warm up, 2) the sympathetic activation and parasympathetic withdrawal necessary to ensure anticipatory metabolic and cardiovascular responses to a physical effort [20], and 3) the mental stress and anxiety associated with competition proximity [21], [22]. This anticipatory HR response was even more pronounced in the junior group (about 8 beats·min^−1^ higher on average) suggesting that senior synchronized swimmers might be better adapted to competition stress due to higher competitive experience and/or specific training. This is in accordance with the conscious processing hypothesis [23], which states that stress affects performance through a process in which anxiety induces a conscious reinvestment of explicit knowledge to control the execution of the skill and, paradoxically, disrupts the automaticity of performance. This limitation in performance has been consistently reported in relation to self-focused (internal) attention [24], [25]. Internal attentional focus constrains the motor system by interfering with natural control processes, whereas an external focus seems to allow automatic control processes to regulate the movements associated with optimal performance and is typically found in expert-level athletes [26]. An alternative explanation is that in competitive situations novice performers are highly motivated to do well and this leads to a tendency to focus on the process of performing [27]. Thus, junior swimmers, who may be more aware of the importance of precise skill execution, would have attempted to ensure success by more consciously monitoring their performance. On the contrary, the attainment of a higher skill level (i.e. typically in seniors swimmers), would be associated with a greater automaticity in performing motor acts, related to a lower metabolic energy cost for achieving the task goal, thus reducing attentional demands and using an energy-efficient preferred mode [28]–[30], which in turn would imply a blunted HR pre-activation response. Present results are consistent with previous findings in highly skilled golfers in comparison to novice players [31]–[33]. In short, competitive experience and years of training would have an effect on swimmers’ anticipatory HR pre-activation related to higher levels of automaticity in task performance, lower levels of anxiety prior to competition, and a different pattern of attentional focus. A unique aspect of SS is the frequent and often lengthy breath holding (BH) periods while performing high-intensity exercise underwater. A key finding of this study is that the main cardiovascular response to BH (i.e. bradycardia) was powerful enough to counteract the HR response during the BH phases of intense exercise (figure 2). It is well known that BH has marked effects on blood pressure (BP), cardiac output, and HR during and after dynamic exercise, which do not seem primarily induced by the resulting hypoxia, where the respiratory arrest *per se* is essential for these cardiovascular responses [34], [35]. Dynamic apnea, as observed for instance in free diving competitions, has shown to induce an increase in BP, which stimulates the circulatory baroreceptors provoking bradycardia, peripheral vasoconstriction and reduced cardiac output, thus decreasing oxygen uptake [36]. These responses would result in restricted muscle metabolism and blood flow redistribution to areas where demands are greatest in order to allow sustained function [37]. However, BH epochs may respond not only to underwater immersion, but also to face water immersion [38], as well as to isometric contraction of the core muscles causing a Valsalva effect.

Thus, during SS, the diving response appears to be powerful enough to override the HR response to exercise during apnea. Cardiac output is expected to be reduced throughout dynamic apneas, largely due to bradycardia, whereas the systemic vascular resistance would increase [36]. These cardiovascular responses are obviously interplaying during water immersion and BH phases of SS routines due to intense exercise combined with BH, which would produce a rapid development of hypercapnia and hypoxia [35]. While apnea and facial immersion increase the parasympathetic tone causing HR reduction [34], [39], exercise increases sympathetic stimulation of the heart [3] and increases HR. So when the swimmer starts holding breath during the routines, both inputs compete with each other for control of HR [40] and O_2_ flow to the exercising muscles, though the O_2_ conservation diving response would finally prevail until the swimmer is able to breathe again.

The observed periods of bradycardia in our swimmers, who reached minimum HR of 75–95 beats·min^−1^ on average (mean 46% HR reduction) were similar to those found during dynamic apnea diving [35], [40], [41], and in SS during training exercises [3], [5], [13], [42], and was more pronounced than the 38% relative HR reduction observed during face immersion during low-intensity (80 and 100 W) cycling exercise [36], [43]. We should take into account that while synchronized swimmers perform several movements combining isometric and intense dynamic exercise, in most previous studies the subjects performed low-intensity, steady-state exercise with face immersion in water to elicit the diving response. Hence, the combination of movements in the pool, with sequential or simultaneous jumps, strokes, acrobatics, and diving across the SS routines, yields HR values proportional to exercise intensity but also induces a bradycardic response similar to diving alone [15] or combined with low intensity exercise [35].

In all routines, high HR_peak_ values indicated a very intense activation of the cardiovascular system to ensure the high-energy turnover in the exercising muscles. These values are higher than previously reported by Jamnik (1987) [3] who found HR_peak_ values ranging from 161 to 180 beats·min^−1^ during solo, duet and team training routine exercises, as well as compared to 178.0±4.2 and 179.5±4.9 beats·min^−1^ during technical and free duets shown by the same two elite swimmers reported by Pazikas et al. (2005) [13]. They are also higher than those observed during a simulated training routine consisting on standard SS maneuvers executed while swimming in straight lines up and down the pool during 4.5 minutes (176±7 beat·min^−1^) [6]. We found no references in literature that can be directly comparable with present results. During competition, HR rapidly increases showing an underlying pattern of exponential increase to asymptotic maximal levels with marked bradycardic episodes (figure 2). This suggests that BH oxygen conservation mechanisms do not prevent the activation of the cardiorespiratory system to provide energy for the exercising muscles despite blunting the HR response during the periods of apnea.

The fact that we found no differences in HR_peak_ between juniors and seniors is likely to be an indication that all routines were performed at maximal intensity by all swimmers despite the observed differences in performance as quantified by final competition scores. Interestingly, no differences in HR_peak_ among the different routine programs were noted despite the wide range of exercise duration (roughly 2 to 4 min), in contrast with significant differences in recovery HR, La_peak_, and RPE, which would support the shared concepts that solo and duet routines are physically more demanding than team routines, and that free routines are generally more so than technical programs.

With respect to HR recovery parameters (HR_post3_ and HR_post5_), the FT routines show a slower off-kinetics then TS. We can only propose a plausible explanation to this observation, which is the lower average cardiorespiratory fitness in team swimmers as compared to soloists (all of them World medalists in our sample). This would be in accordance with previous findings showing that a lower HR during recovery is a specific adaptation in trained synchronized swimmers [15]. Likewise, since both category groups exhibited similar HR off-dynamics, junior and senior swimmers in this study seemed to be similarly adapted to SS training. Whether this adaptation derived from similar levels of general cardiorespiratory fitness or to an enhanced ability to breath hold as a specific feature of SS training adaptation [15] could not be elucidated.

In summary, cardiovascular demands of all SS competitive routines, which are described for the first time during actual competition in a large number of subjects, can be best described as very high, regardless of its duration and technical content. Since the HR response is largely depending on BH responses, it seems logical to assume that non-specific laboratory tests would not accurately reproduce specific cardiovascular loading and hence miss an important feature of specific adaptation to SS performance. Simulated routines with high technical content in a training environment would seem to be a better approach if these adaptations should be assessed or elicited. However, we must realize that HR–even if a practical and measurable indicator of the cardiorespiratory adaptation to physical effort–is influenced by many physiological factors during this unique combination of intense, finely coordinated exercise, frequent apneic periods, and sudden changes in body position. They call into play simple reactions (e.g. diving reflex, Valsalva reflex) and complex regulatory mechanisms (e.g. brain and muscle perfusion, cardiac output and blood pressure regulation).

### Blood Lactate

Elite synchronized swimmers are exposed to hypoxia because of the combination of BH and vigorous exercise [44]. However, the present results indicated moderate La_peak_ in both age categories, ranging from ∼5 to 13 mmol·L^−1^, with an overall average of 7.3 mmol·L^−1^ (table 5). La_peak_ data from competition are very scarce. Although reports on lactate levels during training are more extensive [3], [6]–[8], [14], only Jamnik et al. (1987) [3] reported an intriguing average of 12.7±1.3 mmol·L^−1^ in five elite swimmers, surprisingly higher than the 7.0±1.3 mmol·L^−1^ when performing the same routine during practice.

The highest La_peak_ values were obtained in free solo and duet programs. These observations can be analyzed in terms of 1) the specific influence of the BH periods, 2) the activation of the glycolytic metabolism in the exercising muscles, and 3) the specific training adaptations.

First, the peripheral vasoconstriction associated with the diving response during the BH periods would reduce the blood supply to the muscles and lower their O_2_ stores and, as a consequence, if the energy turnover in the exercising muscles is sustained or increased, the glycolytic metabolism will be activated and more lactic acid be produced [43], [45], [46]. Homma et al. (1994) [2] showed that the time spent underwater in international competitions was highest in solo (62.2%), duets (56.1%), and teams (51.2%). Then we could speculate that the more reduced peripheral O_2_ delivery due to the longer or more frequent BH times [2], [42], the higher the lactate production due to hypoxemia. This seems consistent with our observation that free solo and duet routines induced the highest La_peak_ values as compared with the team and technical routines. From a mechanistic perspective, moderate lactic acidosis would decrease the affinity of myoglobin and hemoglobin for O_2_, thus facilitating O_2_ diffusion to muscle mitochondria for sustained oxidative phosphorylation during the apneic bouts. Thus, with progressive lactate accumulation during the routines, increased O_2_ supply may be made available, leading to prolongation of oxidative metabolism in parallel with anaerobic glycolysis [37]. Moreover, our findings are in line with previous studies in eupneic aesthetic sports such as rhythmic and sport gymnastic events of shorter duration (∼1.5 min), e.g. competitive aerobic (7.5 mmol·L^−1^) [47], floor exercises in artistic gymnastics (8.5 mmol·L^−1^) [48], but also with longer events (∼4.5 min) such as figure ice skating (7.4 mmol·L^−1^) [49]. Nevertheless, higher average values have been described after competition in disciplines with an intermittent respiration pattern and similar duration, such as 200 m freestyle swimming (10.5 mmol·L^−1^) [50], surf lifesaving (9.0 mmol·l^−1^) [51], and even in competitive dynamic apneas (10.0 mmol·l^−1^), in which apneic duration is essential and needs to be prolonged by any means to increase gas storage or tolerance to asphyxia. In contrast, we noted no difference between our data and those attained by elite underwater hockey players (8.0 mmol·L^−1^) [52]. These results may be explained by the specific training pattern of SS, characterized by frequent and intense bouts of dynamic apnea interspersed by short breaths with relatively low tidal volumes compared with free divers. Such differences may indicate that during eupneic work, part of the lactate produced in the working muscles is rapidly catabolized by the less active muscles and other tissues, or used during recovery to resynthesize glycogen. However during apneic diving, lactate removal from working muscles may be compromised by selective vasoconstriction, and restricted blood flow may lead to considerable regional differences in lactate concentration [37].

Second, we should avoid interpreting the La_peak_ values in terms of the sole variations of its cellular production because lactate in capillary blood samples will reflect the balance between production and catabolism (mainly intracellular and in other organs and less active muscles) [53]. The higher La_peak_ values obtained in FS and FD competitive routines (∼3–3.5 min) suggest a more intense activation of anaerobic glycolysis [14]. Empirically, many coaches and swimmers believe that FS and FD are the most strenuous routine programs. Our data do support this concept, as La_peak_ is highest in free solo and duet. Several hypotheses can be advanced to explain these results. On the one hand, free programs usually start with an underwater sequence which may last in excess of 45 s in the case of more highly placed contestants [44]. In spite of blood flow redistribution, O_2_ stores might be reduced at the onset of the routine and, hence, the working muscles would receive less O_2_ than required due to peripheral vasoconstriction and would then rely more on glycolytic metabolism. On the other hand, the rate of execution of skill elements tends to be higher in the solo event than in duet and team [2]. In fact, in solos, 50% of the technical merit score depend on the execution [1], then not being surprising that this event is composed of more figure parts implying a higher physiological stress than duets (51.9%) and teams (32.2%) [2], [5]. Especially in duets, swimmers generate actions requiring constant fine-tuned synchronization with music and couples [54]. Moreover, the difficulty and order of the figures could have also influenced the course of activation of the glycolytic metabolism in the exercising muscles. We could only speculate that FS and FD routines may have involved harder elements and figures at the start of the routine with the concomitant increase in the workload, which would result in higher lactate formation. This possibility should be addressed in the above mentioned time-motion analysis.

Third, La_peak_ values indicate an equally moderate blood lactate accumulation in juniors and seniors, evidencing a similar role of the anaerobic metabolism in energy delivery during SS, as suggested by previous studies [8], [10]. This may be explained by the fact that both age categories executed similar technical elements during the routines as they were participating in an absolute championship and judged under the same rules, implying the execution of the same mandatory technical figures performed in the same order within a similar time frame [1]. These results suggest similar metabolic training adaptations between both age groups despite the higher training volume of the senior swimmers. Moreover, there are some similarities between our data and those reported by authors who studied the effects of SS training in blood lactate levels, who found a significant decrease in La_peak_ along a season [6], [7]. Training practice seems to produce such adaptations improving effectiveness at both peak and submaximal exercise [55], and could explain the improvements in work economy by promoting greater technique skills.

In short, this study shows a moderate post-routine blood accumulation in elite senior and junior synchronized swimmers, likely to result from the large number of figures and high execution rate [10], paralleled by reduced peripheral O_2_ delivery due to BH periods and the subsequent diving response [2], [42], and an adaptive mechanism to assure central oxygenation. At this time, one may only speculate on the progressive development of an adaptive metabolic response in synchronized swimmers to repetitive apneas, which should be explored using longitudinal studies.

### Rate of Perceived Exertion

RPE has been defined as the subjective intensity of effort, strain, discomfort, and/or fatigue that is experienced during physical exercise [56]. It has been suggested that the inputs for perceived exertion can be categorized into those of central and peripheral origin [57]. Central factors linked to RPE are the sensations primarily associated with the cardiorespiratory system resulting from tachycardia, tachypnea, and dyspnea. Sensory input for RPE of local origin produce the sensation of strain in the working muscles and joints.

The CR-10 category ratio scale developed by Borg [58] appears to be one of the best choices regarding its psychometric characteristics and criterion-related validity [16]. However, RPE scales have barely been used during real competition in short-duration events, and never in SS. Only one study reported RPE (6–20 scale) during an international-level taekwondo competition and found near-maximal HR, high blood lactate levels, and increases in competitors’ RPE across combats [59]. Mean RPE values in the present study ranged from 6.6 (TT) to 8.1 (FD), with quite large inter-individual range of variation (table 5). These scores corresponded to “very strong” to “extremely strong” verbal-anchored levels, with only 3% of the swimmers reaching the absolute maximum intensity (i.e. 10+). Contrarily to HR and La_peak_ levels, RPE values were significantly higher in juniors than in seniors, hence indicating that seniors perceived their performance to be less strenuous. This can be explained by the greater number of years in training and the superior competitive experience in the senior group. This concept is supported by the observation that, while no differences were noted among routines in the junior group, FS and FD routines elicited the highest scores and team routines the lowest in the senior group, and corresponded well to La_peak_ values. In fact, RPE was positively correlated with La_peak_ (R = 0.26), particularly when controlling for age category (R = 0.55). On the one side, based on a recently published meta-analysis [16], RPE scores (CR-10) have been found to be poorly correlated both with HR or blood lactate (mean R = 0.47 and 0.42, respectively), even if the mode of exercise used in previous studies were mostly progressive or intermittent running, walking, or swimming.

### Performance and Physiological Correlates

The relationship between cardiac parameters and performance showed that a higher skill level was associated with a lower anticipatory HR activation and lower levels of bradycardia, with subsequent higher HR range of variation. These relationships are consistent with the notion that the attainment of a proficient level of expertise in SS is related to an improvement of motor automaticity and reduced attentional demands [26], [60], and also to specific physiological responses to apnea training, as suggested by previous studies [52], [61].

Lower anticipatory HR activation, which has been reported in tasks with high external attentional focus, was associated to performance improvements in self-paced sport activities such as rifle and pistol shooting, archery and golf. Our findings are in line with these results. The observed negative correlation between HR_pre_ and performance would reflect decreased afferent inputs to the brain and would result in more effective external focusing of attention and superior performance [62], [63]. Moreover, it appears that juniors, who achieve higher HR anticipatory activation, were putting greater attentional effort to the routine tasks (i.e. internal attentional focus) than seniors, although resulting in lower levels of performance.

A second explanation for increased anticipatory HR activation in the junior swimmers would rather reflect differences in cardiorespiratory responses. On the one side, the anticipatory tachycardic response and hyperventilation may be effective in preparing the body (particularly the O_2_ delivery system) for maximal effort. On the other side, an elevated metabolic rate would further reduce the limited O_2_ stores during apnea. As discussed before, the O_2_-conserving effect of the diving response is explained by a reduction in cardiac output and a redistribution of peripheral blood flow. A decrease in cardiac output during apnea would reduce the pulmonary O_2_ uptake [43], [64]. Thus, during apnea, the rate of arterial O_2_ desaturation is affected by factors related to the size of the O_2_ stores at the beginning, and to the rate of O_2_ usage during exercise [41]. Since the anticipatory HR response is thought to increase the cardiac output before starting the exercise, this would consequently increase the rate of O2 depletion and could limit aerobic performance. This is also consistent with our observation that junior swimmers have higher anticipatory HR pre-activation and lower performance.

On the other hand, we noted an inverse relationship between the level of bradycardia and HR range of variation with performance. It can be hypothesized that a more pronounced bradycardic response–and hence lower HR_min_ and higher HR_range_–may be related to more prolonged BH periods in higher rated routine exercises or to a sharper decrease in HR as a consequence of the increased O_2_ conservation effect in the more experienced swimmers [15]. Bradycardia is an essential protective reaction of the cardiac system aimed at economically managing O_2_ levels during BH [65]. The economical use of O_2_ results from lowered myocardial O_2_ demands causing a decrease of the cardiac output [66]. It is well known that long-term apnea training increases hematocrit, erythropoietin concentration, hemoglobin mass, and lung volumes [39], [67], [68] indicating adaptation to hypoxia. An augmented diving response is associated with a reduced rate of arterial desaturation and a reduced rate of depletion of the lung O_2_ stores during apnea at both rest and exercise, which is thought to reflect the O_2_-conserving effect of the human diving response [38], [43], [64], [69]. In SS this mechanism aims to maintain the O_2_ delivery in support of the most vital functions of heart, brain, and lungs. Our results are in accordance with the observations of pronounced bradycardia in professional skin divers [38], and underwater hockey players [52], suggesting that their exaggerated diving response and superior apneic ability is at least in part a product of their apnea training.

Globally, the fact that the multivariate model included two HR parameters (HR_pre_ and HR_min_) and explained 26% of variability in performance (TCS) supports the concept that an augmented diving response is associated to superior performance in SS. However the conclusion that an augmented diving response is beneficial for SS performance clearly requires further study.

### Conclusions

Cardiovascular responses during competition are characterized by intense anticipatory pre-activation and rapidly developing tachycardia up to maximal levels with interspersed periods of marked bradycardia during the exercise bouts performed in apnea. Moderate blood lactate accumulation appears to be related to the number of figures, execution rate, apneic periods and exercise duration, and suggests an adaptive metabolic response as a result of specific training adaptations attributed to influence of the diving response in synchronized swimmers. Competitive routines are perceived as very to extremely intense by all swimmers, likely reflecting not only the absolute exercise demands but also their previous experience and expectations. HR anticipatory activation and bradycardic appear to be related to the variability of performance in SS, which seems to be associated to more pronounced bradycardic response. However, the role of BH and diving in the physiological response to very intense dynamic exercise warrants further investigation.
